# Close of Lectures

**Published:** 1856-09

**Authors:** 


### EDITORIAL AND MISCELLANEOUS.

### CLOSE OF LECTURES.

The Course of Lectures, in the Atlanta Medical College, will close with the last of August, to be resumed again at the regular period, the first of May, 1857; and for the benefit of those who are grossly ignorant, or disposed to wilfully misrepresent, we state that there is only *one* Session or Course of Lectures in this institution *annually*, and degrees are only conferred *once* a year.

The principles of the Institution and the requirements, being the same with those of the highest in the land; and without going into other grounds of objection, of which we occasionally hear, such as authoritative declarations, upon the part of jealous and self-important professors in other schools "that we cannot teach Anatomy in the summer," we would merely point our remaining enemies to the fate of their *leader*, poor Ramsay, (whose highest ambition seemed to be to crush the Atlanta Medical College,) and warn those who continue their dishonorable opposition to the interests of this School, that unless the doctrine of a special providence fail, a heavy retribution will yet fall upon their selfish heads.

At last, however, the *number and character* of those who have been engaged in this war upon us, has grown "small by degrees, and beautifully less," and the hostile demonstrations which are occasionally exhibited, may be looked upon, merely, as the scattering shots of "*inglorious retreat.*" We have no hesitation in asserting that we shall exhibit to the country the most conclusive evidence that the necessary facilities for medical education are furnished here; in the character and qualifications of those whom we shall send out as graduates.

There are now quite a large number who have thus become connected with us, and whose interests are identified with ours, and to whom we are looking with the most confident anticipations, as sources from which their Alma Mater is to derive credit and honor, as the result of their successful career. And it is with no little pleasure that we are expecting to aid in building up science and the just reputation of many who have been students here, by *recording* the results of their investigations, when they shall have engaged in the practical pursuit of their profession. And we cannot too strongly urge upon them the importance of careful observation and a faithful record of their practical professional life, not being deterred by the fear of criticism from contributing their aid to the advancement of science; but, having by earnest and persevering labor, acquired a knowledge of their calling, or, in other words having *learned a fact* in Atlanta, *know it* as certainly as if it had been acquired in London or Paris, for as there is "no royal road to learning," so there is no royal *seat* of learning; Science at the *South*, being equally Science, North, East or West.

To all, then, who have sustained the relation of student to this Institution, and especially to those who are about setting out upon life as physicians, we would say, proceed cautiously, yet boldly, and with honest and earnest purpose, to the discharge of the weighty responsibilities you have assumed—maintain a "conscience void of offence, both toward God and toward man," and with energy and perseverance, you cannot fail to reap a rich reward here and hereafter. And in conclusion, as our connection as teacher and student is about being dissolved, without indulging in the set terms, or honied phrases of a formal farewell, but as the out-going of the overflowing hearts of the Faculty, we have to return our sincere thanks to the Class for their uniform courtesy, kindness and consideration, and for the faithful assiduity with which they have devoted themselves to their duties; and while it is a relief, of course, that our labors are suspended, it is in all honesty coupled with regret that such pleasant associations are about to be sundered, and hoping that all who have had any connection with us, may meet with full success in life, and that they may *deserve* even more than they receive, we leave with them, as a stimulus to exertion, and a parting sentiment in relation to our noble profession, "If you look to its antiquity, it is most ancient—if to its dignity, it is most honorable—if to its scope, it embraces a world in its arms."

